# Safety and efficacy of irinotecan, oxaliplatin, and capecitabine (XELOXIRI) regimen with or without targeted drugs in patients with metastatic colorectal cancer: a retrospective cohort study

**DOI:** 10.1186/s12885-022-09889-3

**Published:** 2022-07-21

**Authors:** Xiu Liu, Kai Ou, Xiaoting Ma, Lizhen Gao, Qi Wang, Haizeng Zhang, Lin Yang

**Affiliations:** 1 Department of Medical Oncology, National Cancer Center/National Clinical Research Center for Cancer/Cancer Hospital, Chinese Academy of Medical Sciences and Peking Union Medical College, No.17 Panjiayuannanli, Beijing, 100021 China; 2 Department of Medical Oncology, Beijing Chaoyang Huanxing Cancer Hospital, Beijing, 100023 China; 3 Department of Medical Oncology, Beijing Chaoyang District Sanhuan Cancer Hospital, Beijing, 100122 China; 4 Department of Colorectal Surgery, State Key Laboratory of Molecular Oncology, National Cancer Center/National Clinical Research Center for Cancer/Cancer Hospital, Chinese Academy of Medical Sciences and Peking Union Medical College, No.17 Panjiayuannanli, Beijing, 100021 China

**Keywords:** Colorectal cancer, Triplet chemotherapy, Irinotecan, Oxaliplatin, Capecitabine

## Abstract

**Background:**

Five-fluorouracil, folinic acid, oxaliplatin and irinotecan (FOLFOXIRI) regimen is used as the first-line treatment for metastatic colorectal cancer (mCRC). The use of capecitabine, an oral fluoropyrimidine pro-drug, is feasible and safe; hence, it provides an interesting alternative to 5-fluorouracil in the abovementioned regimen. This study aimed to evaluate the efficacy and safety of capecitabine, oxaliplatin, and irinotecan (XELOXIRI) regimen use with or without targeted drugs in Chinese patients with mCRC.

**Methods:**

We conducted a retrospective, longitudinal cohort study of patients with mCRC who received XELOXIRI regimen with or without targeted drugs (bevacizumab or cetuximab) every 2 weeks between January 2017 and November 2019 at the National Cancer Center/Cancer Hospital, the Chinese Academy of Medical Sciences, and Peking Union Medical College. Treatment efficacy was assessed by investigators by evaluating the objective response rate (ORR) and disease control rate (DCR). Overall survival (OS) was assessed using Cox proportional hazards models. The adverse events were also analyzed.

**Results:**

Sixty-one consecutive patients were examined and followed up for survival. As of November 8, 2021, the median follow-up time was 35.4 months. Disease progression and death occurred in 50 (82%) and 38 (62%) patients, respectively. The median treatment duration of XELOXIRI with or without bevacizumab or cetuximab was 10 cycles (range, 1–12 cycles). The median OS and PFS were 32.2 months (95%CI [24.8–39.6]) and 9.3 months (95% CI [8.1–10.5]), respectively. The ORR of 48 patients with measurable lesions was 70.8%, and the DCR was 89.6%. *RAS*/*BRAF* wild-type (HR 0.39; 95% CI [0.16–0.96], *p* = 0.04) and metastatic organs > 2 (HR 3.25; 95% CI [1.34–7.87], *p* = 0.009) were independent prognostic factors for OS. The incidence of any grade of adverse events (AEs) was 96.7% (59/61). Grade ≥ 3 AEs included neutropenia (19.7%), leukopenia (9.8%), diarrhea (3.3%), vomiting (3.3%), febrile neutropenia (1.6%), and thrombocytopenia (1.6%). No treatment-related death occurred.

**Conclusion:**

The use of the XELOXIRI regimen with or without a targeted drug was effective, with a manageable toxicity profile in Chinese patients with mCRC.

**Supplementary Information:**

The online version contains supplementary material available at 10.1186/s12885-022-09889-3.

## Background

Over the last decade, the triplet chemotherapy combining irinotecan, oxaliplatin, and 5-fluorouracil (FOLFOXIRI) with or without monoclonal antibodies (bevacizumab or cetuximab) as the first-line treatment of metastatic colorectal cancer (mCRC) has shown a significantly improved overall response rate (ORR), progression-free survival (PFS), overall survival (OS), and R0 resection rate of liver metastases than standard doublet chemotherapy regimens [1–11]. Since 2015, FOLFOXIRI plus bevacizumab has been listed as a first-line treatment option for fit patients with mCRC in several clinical guidelines worldwide [12–15]. However, the use of this regimen is also characterized by high toxicity, even in well-designed clinical trials, most of which were conducted in European and American populations; additionally, there is no uniform dose level of the components of this regimen. Few studies in China and Japan have reported that Asian populations can tolerate lower doses of the component drugs in the FOLFOXIRI regimen, especially the dose of irinotecan (which is recommended to be 150 mg/m^2^). Moreover, indirect comparisons have shown a higher incidence of neutropenia in Asian patients than in Western patients [16, 17]. In a phase I dose-escalation study conducted in China, the maximum tolerated dose of irinotecan was only 150 mg/m^2^ in a single infusion on day 8 [17]. The dose-limited toxicities (DLTs) of irinotecan were diarrhea and febrile neutropenia. The FOLFOXIRI has a limited clinical application, as the original regimen is poorly tolerated, especially in Asian patients; hence, an expert consensus in China has recommended the use of modified FOLFOXIRI [18, 19]. The Chinese modified FOLFOXIRI (cmFOLFOXIRI) regimen consists of intravenous infusions of oxaliplatin 85 mg/m^2^ over 120 min, irinotecan 150–165 mg/m^2^ over 90 min, folinic acid 400 mg/m^2^ over 120 min, and 5-fluorouracil 2,400–2,800 mg/m^2^ over a 46–48 h continuous infusion every 2 weeks. Nevertheless, FOLFOXIRI or cmFOLFOXIRI use is inconvenient, as it necessitates a continuous infusion of 5-fluorouracil, which requires the placement of indwelling central venous catheters and portable infusion pumps.

Capecitabine, an oral fluoropyrimidine pro-drug that demonstrates a superior safety profile and convenience, can be used as an alternative to 5-fluorouracil [20–23]. The use of the triplet regimen (XELOXIRI, COI, CAPOXIRI, or XELIRINOX), with capecitabine replacing 5-fluorouracil as the fluoropyrimidine backbone with or without antibodies, has been investigated in several phases I and II clinical trials in the first line and in conversion setting with different dose schedules [24–29]. The DLTs were grade 4 neutropenia and grade 3 diarrhea. The incidence of grade 3/4 neutropenia (0–57%) with XELOXIRI use was as high as that with FOLFOXIRI use, while the incidence of grade 3/4 diarrhea (0–40%) seems higher than that of FOLFOXIRI use, especially in patients with mCRC in Western countries [24–29]; this demonstrates the ethnic differences in oral fluoropyrimidine metabolism and tolerability between Western and Eastern Asian populations.

This study aimed to retrospectively evaluate the efficacy and safety of XELOXIRI use with or without targeted drugs in Chinese patients with advanced colorectal cancer.

## Methods

### Study design and population

This was a retrospective, longitudinal cohort study conducted at the National Cancer Center/Cancer Hospital, the Chinese Academy of Medical Sciences, and Peking Union Medical College.

From January 1, 2017, to November 30, 2019, we examined eligible patients who developed a histologically confirmed adenocarcinoma of the colon or rectum, with the first occurrence of metastatic disease deemed unresectable at the age of at least 18 years and the presence of measurable and/or assessable lesions according to RECIST1.1 criteria. Furthermore, we included patients who had initiated therapy with XELOXIRI alone or combined with bevacizumab or cetuximab every 2 weeks for mCRC and had at least one visit to the study center. Major exclusion criteria included patients with previous chemotherapy or targeted therapy history (excludes patients with recurrence and metastasis more than 6 months away from adjuvant or neoadjuvant chemotherapy) and other malignant tumours in the past 5 years (except for cervical carcinoma in situ, cutaneous squamous or basal cell carcinoma treated for radical purposes).

We collected data on the following demographic and clinical characteristics at baseline: primary cancer site, sites of metastases, genetic mutation status, treatment type, dosing and drug dose modifications, tumour response, treatment after XELOXIRI use, survival, and patient conditions while on treatment, as documented in our medical record system. PFS was defined as the time period from the date of chemotherapy initiation to the date of imaging-confirmed disease progression or the death from any cause before disease progression. Disease-free survival (DFS) was defined as the time period from the day of R0 resection to imaging-confirmed disease progression or death due to any cause before cancer recurrence or the appearance of a second primary cancer. OS is defined as the time period from the date of chemotherapy initiation to death from any cause. ORR, DCR, PFS and DFS were evaluated by the investigator retrospectively. This study was approved by the Ethics Committee of the National Cancer Center/Cancer Hospital, Chinese Academy of Medical Sciences, and Peking Union Medical College.

### Statistical analysis

Descriptive statistics were used to analyze demographic as well as clinical efficacy and safety data at baseline and during follow-up. The measurement data were analyzed using the *t*-test and count data using the χ2 test. The Wilcoxon rank-sum test was used for the comparison of grade data. The ORR was defined as the proportion of patients with the best response of complete response (CR) or partial response (PR), and disease control rate (DCR) was defined as the proportion of patients with the best response of CR, PR, or stable disease (SD) according to RECIST1.1 criteria. Survival analysis was performed using the Kaplan–Meier method to estimate the median and 95% confidence interval (CI) of the incidence of events. The log-rank test was used for subgroup analysis. Cox regression analysis was used to evaluate the impact of research factors on survival or risk rate. All statistical tests were two-sided, and *p*-values < 0.05 were considered statistically significant. Statistical analyses were performed using SPSS 22.0 software (IBM Corp., Armonk, New York, USA).

## Results

### Patient characteristics

Patient clinical and pathological features are shown in Table 1. This study included 61 patients, 38 (62.3%) of whom were men. The median age was 50 years (range, 26–70 years). In 19(31.1%) patients, the primary tumour was in the right colon (from the cecum to the transverse colon). Forty-two (68.9%) patients had left-sided tumours, including 17 (27.9%) and 25 (41.0%) patients whose primary tumours were in the colon (from the splenic flexure to the sigmoid colon) and rectum, respectively. Moreover, 56 patients (91.8%) had synchronous distant metastases at diagnosis, 14 (23%) and 11 (18%) of whom had peritoneal metastases and more than two metastatic sites, respectively. Genetic mutation status was available in 54 patients. We found that 23 (37.7%) patients had *RAS* mutation, 12 (19.7%) patients had *BRAF*^V600E^ mutation, and 19 (31.1%) patients had *RAS*/*BRAF*^V600E^ wild-type (WT). No known high microsatellite instability (MSI-H) was present.

**Table 1:** Patient characteristics

N (%)	All	XELOXIRI	XELOXIRI + BEV	XELOXIRI + CET
	61 (100)	39 (63.9)	18^a^ (29.5)	4 (6.6)
Sex				
Male	38 (62.3)	27 (44.3)	9 (14.8)	2 (3.3)
Female	23 (37.7)	12 (19.7)	9 (14.8)	2 (3.3)
Age				
Median (range)	50 (26–70)	60 (27–70)	38 (36–66)	29.5 (26–46)
≤ 65 years	52 (85.2)	31 (50.8)	17 (27.9)	4 (6.6)
> 65 years	9 (14.8)	8 (13.1)	1 (1.6)	0
ECOG				
0	20 (32.8)	15 (24.6)	4 (6.6)	1 (1.6)
1	41 (68.3)	24 (39.3)	14 (23.0)	3 (4.9)
Primary location				
Right colon	19 (31.1)	13 (21.3)	6 (9.8)	0
Left colon	42 (68.9)	26 (42.6)	12 (19.7)	4 (6.6)
Colon	17 (27.9)	6 (9.8)	9 (14.8)	2 (3.3)
Rectum	25 (41.0)	20 (32.8)	3 (4.9)	2 (3.3)
Surgery on primary tumour				
Yes	7 (11.5)	4 (6.6)	3 (4.9)	0
No	54 (88.5)	35 (57.4)	15 (24.6)	4 (6.6)
(Neo)adjuvant chemotherapy				
Yes	6 (9.8)	3 (4.9)	3 (4.9)	0
No	55 (90.2)	36 (59.0)	15 (24.6)	4 (6.6)
(Neo)adjuvant radiotherapy				
Yes	1 (1.6)	1 (1.6)	0	0
No	60 (98.4)	38 (62.3)	18 (29.5)	4 (6.6)
Time to metastasis				
synchronous	56 (91.8)	35 (57.4)	17 (27.9)	4 (6.6)
Metachronous	5 (8.2)	4 (6.6)	1 (1.6)	0
Metastasis sites				
Liver	41 (67.2)	27 (44.3)	11 (18.0)	3 (4.9)
Liver-only	15 (24.6)	9 (14.8)	5 (8.2)	1 (1.6)
Lung	18 (29.5)	12	6 (9.8)	0
Lung-only	5 (8.2)	4 (6.6)	1 (1.6)	0
Peritoneum	14 (23.0)	8 (13.1)	5 (8.2)	1 (1.6)
Peritoneum-only	7	3(4.9)	3(4.9)	1(1.6)
Numbers of metastatic organs				
1	32 (52.5)	20 (32.8)	10 (16.4)	2 (3.3)
2	18 (29.5)	13 (21.3)	4 (6.6)	1 (1.6)
> 2	11 (18.0)	6 (9.8)	4 (6.6)	1 (1.6)
*RAS* and *BRAF* status				
* RAS* mutant	23 (37.7)	17 (27.9)	6 (9.8)	0
* BRAF* mutant	12 (19.7)	4 (6.6)	8 (13.1)	0
* RAS*/*BRAF* wild-type	19 (31.1)	13 (21.3)	2^a^ (3.3)	4 (6.6)
Missing data	7 (11.5)	5 (8.2)	2 (3.3)	0
Primary tumour site and *RAS*/*BRAF* status				
Right and *RAS*/*BRAF* wild-type	2 (3.3)	1 (1.6)	1 (1.6)	0
Right and *RAS* mutant	11 (18.0)	8 (13.1)	3 (4.9)	0
Right and *BRAF* mutant	2 (3.3)	2 (3.3)	0	0
Left and *RAS*/*BRAF* wild-type	17 (27.9)	12 (19.7)	1 (1.6)	4 (6.6)
Left and *RAS* mutant	12 (19.7)	9 (14.8)	3 (4.9)	0
Left and *BRAF* mutant	10 (16.4)	2 (3.3)	8 (13.1)	0
Missing data	7 (11.5)	5 (8.2)	2 (3.3)	0

*BEV* Bevacizumab, *CET* Cetuximab, *ECOG* Eastern cooperative oncology group, *NA* Not available, *XELOXIRI* Capecitabine, oxaliplatin and irinotecan

^a^ One patient received XELOXIRI + CET therapy for three cycles after nine cycles of XELOXIRI + BEV

### Treatment

We found that 39, 4, and 17 patients received XELOXIRI alone, XELOXIRI plus cetuximab, and XELOXIRI plus bevacizumab, respectively; moreover, one patient initially received a XELOXIRI-bevacizumab combination, which was later switched to a XELOXIRI-cetuximab combination (Table 1). The dose schedule of XELOXIRI was as follows: irinotecan infusion for 1 h (at a dose of 120–170 mg/m^2^), then oxaliplatin infusion for 2 h (at a dose of 70–100 mg/m^2^), and oral capecitabine (at a dose of 600–1000 mg/m^2^ twice daily) for 1–7 days every 2 weeks. For patients treated with targeted drugs, bevacizumab (5 mg/kg) or cetuximab (500 mg/m^2^) was administered on the same day ahead of XELOXIRI treatment, every 2 weeks.

### Efficacy

As of November 8, 2021, all patients were followed up for survival; the median duration of follow-up was 35.4 months (range, 3.9–52.9 months). Disease progression and death occurred in 50 (82.0%) and 38 (62.3%) patients, respectively. The median OS was 32.2 months (95% CI [24.8–39.6]), and the 1-, 2-, and 3-year OS rates were 84.6%, 55.1%, and 38.6%, respectively. The median PFS was 9.3 months (95% CI [8.1–10.5]), and the 1-year PFS rate was 35.0%. Regarding tumour response, assessed by investigators, none of the 48 patients with measurable lesions achieved CR, whereas 34 achieved PR, resulting in an objective response rate of 70.8% (95% CI [64.1%-77.5%]). The DCR was 89.6% (95% CI [82.9%-96.3%]) (Table 2). Of 15 patients with only liver metastases, 13 achieved PR and 2 had SD. The ORR and DCR were 86.7% and 100%, respectively. Twenty-four patients (39.3%) underwent surgery, including 16 (26.2%) patients who underwent combined resection of primary tumour and metastases and achieved no evidence of disease (NED). Five patients with synchronous unresectable distant metastases had only the primary tumour resected. Another three patients had the liver metastases resected but with residual. As of November 8, 2021, 13 out of 16 patients with NED had disease progression; 4 patients died, 11 survived, and 1 was lost to follow-up. The median PFS was 10.2 months (95% CI [6.6–13.7]), the median DFS was 5.7 months (95% CI [1.8–9.7]), 1-year DFS rate was 31.3%, and the median OS was not reached.

**Table 2:** Response of patients with measurable disease

Response	All	*RAS* mutant	*RAS* WT	*BRAF*^V600E^ mutant	*RAS*/*BRAF*^V600E^ WT
	*n* = 48	*n* = 18	*n* = 24	*n* = 8	*n* = 16
PR	34(70.8%)	13 (72.2%)	19 (79.2%)	4 (50.0%)	15 (93.8%)
SD	9 (18.8%)	4 (22.2%)	3 (12.5%)	2 (25.0%)	1(6.3%)
PD	4(8.3%)	1 (5.6%)	2 (8.3%)	2 (25.0%)	0
NE	1 (2.1%)	0	0	0	0
NED	1(2.1%)	0	0	0	0
ORR	70.8%	72.2%	79.2%	50.0%	93.8%
DCR	89.6%	94.4%	91.7%	75.0%	100%

Genotype and response rate, Fisher exact probability test (two-sided), *BRAF* ^V600E^ mutant vs. *RAS*/*BRAF*^V600E^ WT, *p* = 0.028

*DCR* Disease control rate, *NED* No evidence of disease, *ORR* Objective response rate, *PR* Partial response, *SD* Stable disease, *WT* wild-type

**Fig. 1:**
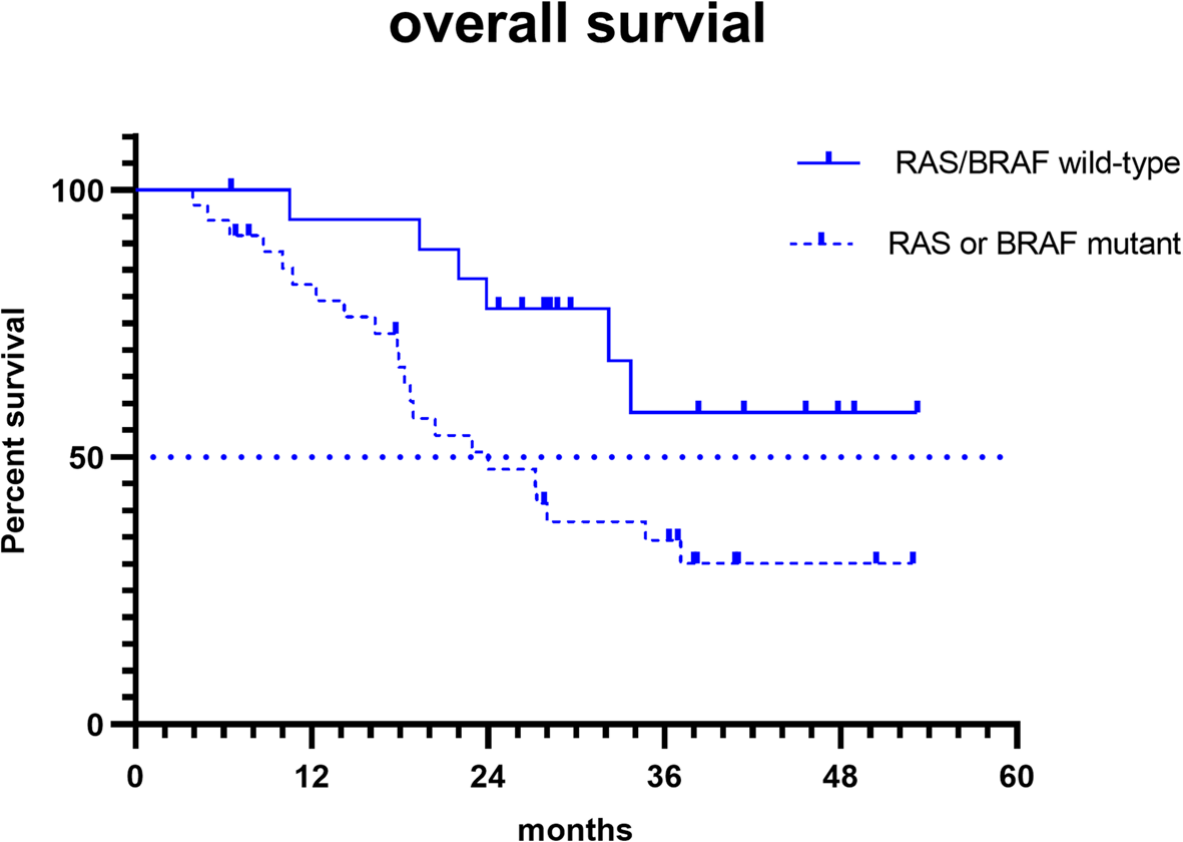
Kaplan Meier survival curves of overall survival (*RAS*/*BRAF*^V600E^ wild-type; *RAS* or *BRAF*.^V600E^ mutant)

**Fig. 2:**
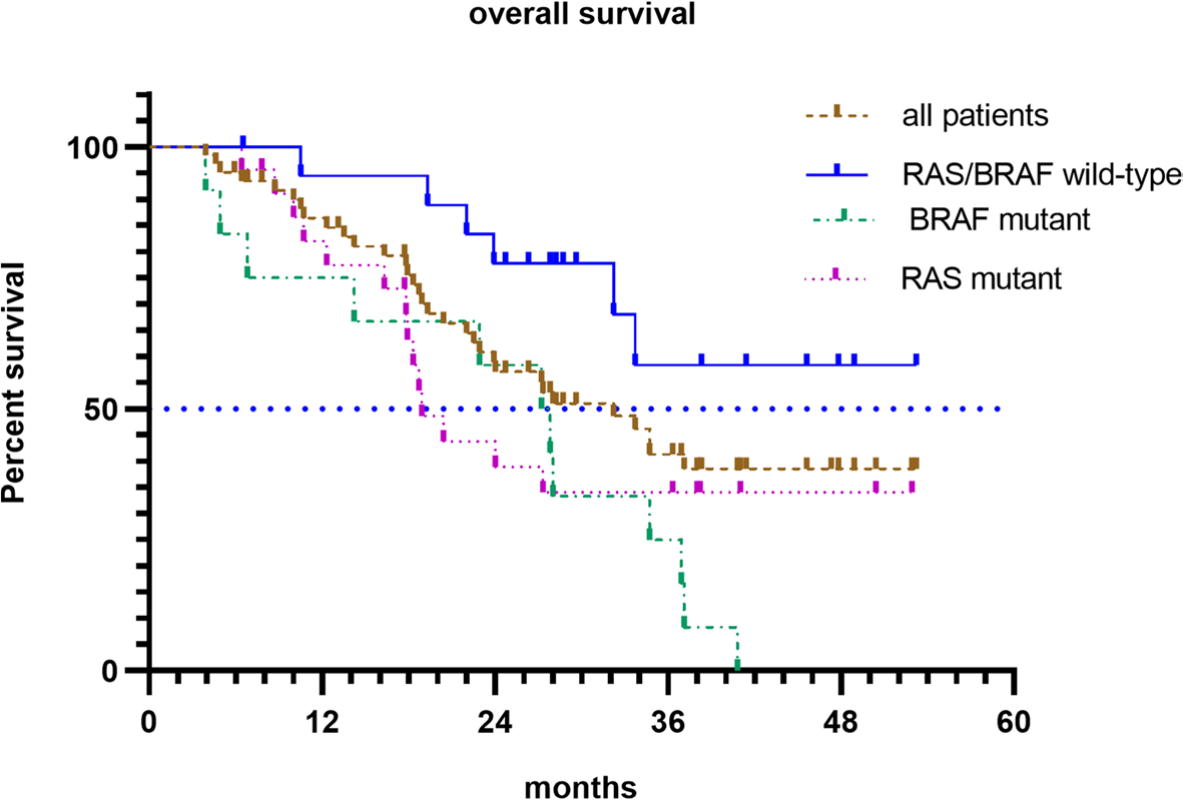
Kaplan Meier survival curves of overall survival (all patients; *RAS*/*BRAF* wild-type; *BRAF* mutant; *RAS* mutant)

**Fig. 3:**
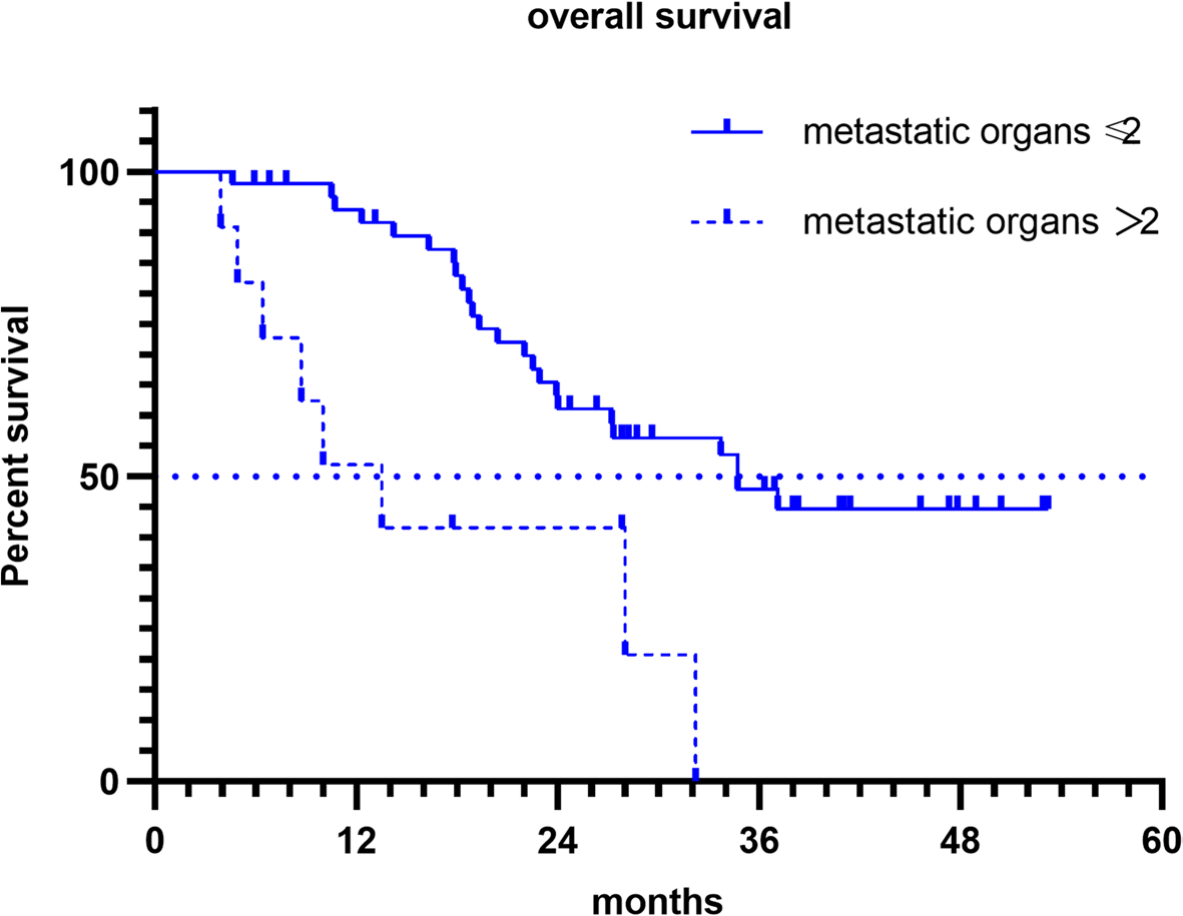
Kaplan Meier survival curves of overall survival (metastatic organs ≥ 2; metastatic organs < 2)

Regarding genetic mutation status, there were significant differences in OS between patients with the *RAS* and *BRAF,* both WT and those with a *RAS* or *BRAF*^V600E^ mutant (not reached vs. 24.0 months; hazard ratio [HR], 0.37; 95% CI [0.15–0.91]; *p* = 0.024) (Fig. 1). Furthermore, the median OS was 18.9 and 28 months for patients with *RAS* and *BRAF*^V600E^ mutants, respectively (Fig. 2). The OS was significantly shorter in patients with more than two metastatic organs than in those with one or two metastatic organs (13.5 vs. 34.7 months; HR 3.46; 95% CI [1.02–11.8]; *p* = 0.001) (Fig. 3). A significant improvement in OS was also observed between patients who achieved NED and those who did not (not reached vs. 28.0 months; HR 0.35; 95% CI [0.16–0.77]; *p* = 0.04). Baseline neutrophil/lymphocyte ratio (NLR) values were available in 52 patients. The median NLR was 2.98 (0.87–31.81); moreover, no significant differences in PFS (HR 1.22; 95% CI [0.67–2.22]; *p* = 0.51) and OS (HR 1.21; 95% CI [0.58–2.50]; *p* = 0.62) were found between patients with NLR ≥ 3 and NLR < 3.

In the multivariable model, *RAS*/*BRAF* WT (HR 0.39; 95% CI [0.16–0.96], *p* = 0.04) and more than two metastatic organs (HR 3.25; 95% CI [1.34–7.87], *p* = 0.009) were independent prognostic factors for OS (Supplementary Fig. 1).

### Tolerance and safety

The median number of treatment cycles of XELOXIRI with or without bevacizumab or cetuximab was 10 (range, 1–12). The main reasons for discontinuing XELOXIRI therapy were surgery (42.6%), switching to maintenance therapy (capecitabine with or without bevacizumab) (14.8%), drug toxicity (6.6%), disease progression (19.7%), loss to follow-up (8.2%), receiving other location therapies (ablation [1.64%] and radiotherapy [4.92%]).

During treatment, 19 patients (31.2%) underwent drug discontinuation, of whom 10 (16.4%), 5 (8.2%), 1 (1.6%), 2 (3.3%), and 1 (1.6%) patients discontinued irinotecan, oxaliplatin, capecitabine, both oxaliplatin and irinotecan, and both irinotecan and capecitabine, respectively. The mean and median dose intensities of oxaliplatin in the combined treatment regimen were 36.95 mg/m^2^/week and 37.31 mg/m^2^/week, respectively. Both the mean and median dose intensities of irinotecan were 71.41 mg/m^2^/week. The mean and median dose intensities of capecitabine were 5599.75 mg/m^2^/week and 5599.75 mg/m^2^/week, respectively (Table 3).

**Table 3:** Treatment exposure

Reasons for discontinuing XELOXIRI	*n* = 61 (%)
Surgery	26 (42.62%)
Maintenance treatment	9 (14.75%)
AEs	4 (6.56%)
Disease progression	12 (19.67%)
Loss to follow-up	5 (8.20%)
Radiotherapy	3 (4.92%)
Ablation	1 (1.64%)
Drug discontinuation	n (%)
Any drug discontinuation	19 (31.15%)
Oxaliplatin	5 (8.20%)
Irinotecan	10 (16.39%)
Capecitabin	1 (1.64%)
Oxaliplatin and irinotecan	2 (3.28%)
Irinotecan and capecitabin	1 (1.64%)
Dose intensity	mg/m^2^/week
Oxaliplatin	
mean dose intensity	36.95
median dose intensity	37.31
Irinotecan	
mean dose intensity	71.41
median dose intensity	71.41
Capecitabin	
mean dose intensity	5599.75
median dose intensity	5599.75

*AE* Adverse event, *XELOXIRI* Capecitabine, oxaliplatin and irinotecan

Table 4 shows the incidences of adverse events (AEs) related to the treatment grade. The incidence of all grades of AEs was 96.7%, and that of grade 3/4 AEs was 32.8%. The most common grade 3/4 AEs included neutropenia (12 [19.7%]), febrile neutropenia (2 [3.3%]), leukopenia (6 [9.8%]), thrombocytopenia (1 [1.6%]), diarrhea (2 [3.3%]), and vomiting (2 [3.3%]). In 8 patients, UGT1A1*6 and UGT1A1*28 genotypes were tested, and 4 had double WT genotypes. Only 1 patient with a heterozygous UGT1A1*28 genotype developed grade 3 neutropenia. No treatment-related death occurred.

**Table 4:** Adverse events

Adverse event	n (%)				
	Any grade	Grade 1	Grade 2	Grade 3	Grade 4
Any AEs	59 (96.7)	58 (95.1)	29 (47.5)	17 (27.9)	3 (4.9)
Decreased appetite	43 (70.5)	43 (70.5)	0	0	0
Nausea	46 (75.4)	32 (52.5)	14 (23.0)	0	0
Vomiting	20 (32.8)	7 (11.5)	11 (18.0)	2 (3.3)	0
Diarrhea	13 (21.3)	9 (14.8)	2 (3.3)	2 (3.3)	0
Stomach ache	6 (9.8)	6 (9.8)	0	0	0
Peripheral neurotoxicity	19 (31.1)	17 (27.9)	2 (3.3)	0	0
Hand-foot skin reaction	10 (16.4)	10 (16.4)	0	0	0
Fatigue	24 (39.3)	23 (37.7)	1 (1.6)	0	0
Rash	4 (6.6)	4 (6.6)	0	0	0
Leukopenia	30 (49.2)	12 (19.7)	12 (19.7)	6 (9.8)	0
Neutropenia	32 (52.5)	11 (18.0)	9 (14.8)	9 (14.8)	3 (4.9)
Febrile neutropenia	2 (3.3)	0	0	1 (1.6)	1 (1.6)
Anemia	14 (23.0)	12 (19.7)	2 (3.3)	0	0
Thrombocytopenia	6 (9.8)	3 (4.9)	2 (3.3)	1 (1.6)	0
Elevated ALT	4 (6.6)	4 (6.6)	0	0	0
Elevated AST	4 (6.6)	4 (6.6)	0	0	0
Anaphylactic reaction	1 (1.6)	0	0	1 (1.6)	0

*AE* Adverse event, *ALT* Alanine aminotransferase, *AST* Aspartate aminotransferase

## Discussion

This retrospective study showed that the use of the triplet XELOXIRI combination with or without an antibody (bevacizumab or cetuximab) as first-line therapy in Chinese patients with mCRC produces a comparable safety and efficacy to that of the FOLFOXIRI regimen.

The median OS of our cohort was 32.2 months, which is higher than that reported in serial phase III trials conducted by the GONO group (22.6–29.8 months) [1–6]. Notably, the proportion of patients harboring the *BRAF*^V600E^ mutant in our cohort was higher (19%) than that in the GONO trials (4.8%-10%)^2, 3, 5–7^. Moreover, 23% of patients in the present cohort had peritoneal metastases; mCRC was most difficult to treat in these patients.

A large amount of evidence indicates that the presence of *BRAF*^V600E^ mutation is related to the poor prognosis of patients with colorectal cancer, which is non-responsive to anti-EGFR treatment [30–38]. The use of cetuximab or panitumumab plus chemotherapy cannot obviously improve the survival of patients with BRAF^V600E^ mutation [39]. In the TRIBE trial, the use of bevacizumab plus FOLFOXIRI showed statistically significant advantages in ORR (56% vs. 42%; OR: 1.82; 95% CI [0.38–8.78]) and OS (19.0 vs. 10.7 months; HR 0.54; 95% CI [0.24–1.20]) compared with the use of bevacizumab plus FOLFIRI in patients with mCRC harboring *BRAF*^V600E^ mutation, which motivated the recommendation of the FOLFOXIRI-bevacizumab combination use by international guidelines. However, in the post-hoc subgroup analyses of the TRIBE2 trial based on the *BRAF*^V600E^mutational status, no significant differences in efficacy were detected between the use of FOLFOXIRI plus bevacizumab and that of the two-drug chemotherapy plus bevacizumab [6]. In this study, 10 of the 12 patients with *BRAF*^V600E^ mutation received XELOXIRI plus bevacizumab therapy; the ORR was 50%, and the mOS of 28 months seemed more promising than that reported in the previous trials^3, 6^ (Supplementary Table 1).

According to a previous study, patients with mCRC having left-sided primary tumours have a better prognosis than those with right-sided primary tumours [40]. Approximately 5% of patients with mCRC have *BRAF*^V600E^ mutation, most of which occur in the right colon. Notably, the proportion of patients with *BRAF* mutations in this study is relatively high, and these patients are not known to have MSI-H; interestingly, most of them have left-sided colon tumours. The left-sided primary tumour may be an explanation for the promising survival of the patients with *BRAF*^V600E^ mutation. However, this result needs to be interpreted carefully and verified further, given the small sample size of this study. Furthermore, it is not surprising that some patients with mCRC having *BRAF*^V600E^ mutations show an indolent clinical course and a relatively favorable prognosis [41].Several studies have demonstrated that 10–20% of patients with *BRAF* mutations can survive for more than 2 years. Moreover, a high level of biological heterogeneity has been revealed in patients with mCRC with *BRAF* mutations (both V600E and beyond V600E), including clinical characteristics, pathological features, and molecular alterations [42–44]. At present, several methods have been established for further stratification of *BRAF* mutations, including subtypes based on signaling mechanisms (classes I, II, and III) [45], molecular consensus subtypes (CMS) (CMS1, CMS2, CMS3, and CMS4) [46], and the transcriptional subtypes of *BRAF*^V600E^ (*BRAF*^V600E^ mutant 1 and *BRAF*^V600E^ mutant 2) [47]. Nevertheless, continuous exploration is needed to reveal the association between molecular profiles and clinical outcomes and to further guide precise treatments using these methods.

Recent international guidelines have recommended the use of NED to evaluate therapeutic efficacy in patients with colorectal liver-limited metastases [8, 48–55]. The use of an effective systemic regimen is the cornerstone for conversions. In this study, the ORR reached 86.7% (13/15) among patients with only liver metastases. Due to the high rate of tumour shrinkage, 26.2% of patients underwent tumour resection and achieved NED and long-term survival. The ORR and R0 resection rates were numerically comparable to those in the FOCULM (95.5% and 55.2%, respectively) and VOLFI (87% and 33%, respectively) trials [8, 9].

NLR has been reported as a poor prognostic factor in several gastrointestinal tumours [56–59]. A retrospective analysis of the TRIBE study found that patients with mCRC with high NLR ≥ 3 had significantly decreased survival benefits (PFS, HR 1.27; OS HR 1.56) compared to patients with NLR < 3 [59]. Inconsistent with this, there was no significant association between NLR and survival in this study. The prognostic value of NLR in patients with mCRC needs to be further evaluated using studies with expanded samples.

So far, XELOXIRI administered every 2 weeks with or without bevacizumab has been investigated on patients with different dose schedules in three phase II and one phase I trials conducted in Western countries; the efficacy was found to be promising [27, 28, 60]; the most common AEs or/ and DLTs were neutropenia and diarrhea (Table 5).

**Table 5:** Similar clinical studies on XELOXIRI

Study and population	Schedule	Irinotecan (mg/m2)	Oxaliplatin (mg/m2)	Capecitabine (mg/m2/d)	Targeted drug	Grade 3–4 AEs	DLT	ORR (%)	PFS (months)	OS (months)
Italy-GONO (2009) Phase 2, *N* = 36	Q2w	165	85	2000	-	Neutropenia (30%), febrile neutropenia (11%), diarrhea (30%)	-	67	10.1	17.9
Italy-ITMO (2007) phase 2, *N* = 38	Q2w	180	85	2000	-	Diarrhea (24%), nausea (16%)	Diarrhea	63	8.5	23.5
Italy (2015) phase 2, *N* = 51	Q2w	180	85	2000	-	Neutropenia (6%), diarrhea (31%), mucositis (4%)	-	62	10.3	22
Spain (2010) phase 1, *N* = 87	Q2w	150	85	2000	-	Neutropenia (27%), diarrhea (11%) and leukopenia (8%)	Neutropenia, diarrhea	66.6	12	27
Canada (2018) phase 1, *N* = 39	Q3w	160	100	1900	-	Neutropenia (56%), diarrhea (15%)	Febrile neutropenia, diarrhea	67	11	25
Japan QUATTRO-II (2021), phase 2, *N* = 9 (Safety lead-in results)	Q3w	200	130	1600	Bev 7.5 mg/kg	Neutropenia (44%)	Febrile neutropenia	89	-	-
Japan (2015) Phase 1, *N* = 12	Q3w	150	100	1700	Bev 7.5 mg/kg	Neutropenia (41%), febrile neutropenia (8%), diarrhea (8%)	Neutropenia	83	15	-
Japan (2017) Phase 1, *N* = 12	Q3w	150	100	1700	Cet 250 mg/m2 qw (Increase the first dose)	Neutropenia (50%), febrile neutropenia (8%), diarrhea (17%)	Neutropenia	83	14.5	-
Japan (2019) phase 1, *N* = 6	Q2W	150	85	2000	-	Febrile neutropenia (2/6)	Febrile neutropenia	-	-	-

*AE* Adverse event, *Bev* Bevacizumab, *Cet* Cetuximab, *DLT* Dose-limited toxicity, *ORR* Objective response rate, *OS* Overall survival, *PR* Partial response, *PFS* Progression-free survival

Italian regimens composed of irinotecan (165 or 180 mg/m^2^), oxaliplatin (85 mg/m^2^), and 1–7 or 2–6 days of capecitabine (2000 mg/m^2^/day) every 2 weeks. The incidences of grade 3/4 neutropenia and diarrhea ranged from 6%-30% and 24%-31%, respectively [27, 28, 60]. Spain’s study showed that a lower dose of irinotecan (150 mg/m^2^) might result in a lower incidence of grade ≥ 3 diarrhea (11%) and a comparable incidence of grade ≥ 3 neutropenia [29] (Table 5). To date, studies concerning the use of triplet drug regimens in Asian populations are limited. Notably, the use of irinotecan-containing regimens seems to increase the incidence of neutropenia in Asian populations. For example, the incidence of grade 3/4 hematologic toxicities with FOLFOXIRI-bevacizumab combination use reached 72.5% in the phase II QUATTRO trial, which was conducted in Japan. However, the incidence of grade 3/4 neutropenia was decreased to less than 50% without compromised efficacy when the dose of irinotecan was modified [8, 61–63]. For XELOXIRI regimens with the 3 weeks schedule, three trials in Japan showed the incidences of grade 3/4 neutropenia ranged from 41%-50% [25, 26, 64] (Table 5). The only published clinical trial from an Asian population using the XELOXIRI regimen (irinotecan 150 mg/m^2^, oxaliplatin 85 mg/m^2^, and 1–7 days of capecitabine 2000 mg/m^2^/day) every 2 weeks was a phase I trial conducted on 6 Japanese patients in the neoadjuvant setting. Grade 3/4 neutropenia occurred in 2/6 (33.3%) patients [24] (Table 5). Similar to our study findings.

Our study had some limitations. This is a retrospective study and thus is inevitably affected by confounding factors. Specifically, the AEs were mainly obtained from patient medical records; hence, these AEs may be missed. Furthermore, due to the small sample size, some patients lacked information related to genetic testing. Moreover, the doses of drugs used in this study were not uniform. However, this study adds efficacy and safety data on the chemotherapy regimens that can be used in the Asian population. We showed that the use of the XELOXIRI regimen, which involves the replacement of 5-fluorouracil with capecitabine, is well tolerated and safe. A phase I/II trial is currently underway to evaluate the appropriate doses, efficacy, safety, and potential predictive or prognostic factors associated with the use of the XELOXIRI-bevacizumab combination regimen as first-line treatment for metastatic colorectal cancer (ChiCTR2000032590, NCT04380103).

## Conclusions

The use of the XELOXIRI regimen with or without a targeted drug was effective, with a manageable toxicity profile in Chinese patients with mCRC.

## Supplementary Information

**Additional file 1: Supplementary Table 1.** Patient survival. **Supplementary Fig. 1.** Cox metanalysis of the impact of research factors on survival or risk rate.

## Data Availability

All data used during this study are available from the corresponding author upon reasonable request.

## Abbreviations

AEs: Adverse events
CR: Complete response
FOLFOXIRI: Irinotecan, oxaliplatin, and 5-fluorouracil
cmFOLFOXIRI: Chinese modified FOLFOXIRI
DCR: Disease control rate
DLTs: Dose-limited toxicities
PR: Partial response
CI: Confidence interval
mCRC: Metastatic colorectal cancer
NED: No evidence of disease
NLR: Neutrophil/lymphocyte ratio
ORR: Objective response rate
OS: Overall survival
PFS: Progression-free survival
SD: Stable disease
WT: Wild-type
XELOXIRI: Capecitabine, oxaliplatin, and irinotecan
