# Supplementary material for: Safety and efficacy of irinotecan, oxaliplatin, and capecitabine (XELOXIRI) regimen with or without targeted drugs in patients with metastatic colorectal cancer: a retrospective cohort study
Source: BMC Cancer. 2022 Jul 21;22:807. doi: 10.1186/s12885-022-09889-3 (PMC9306070; doi:10.1186/s12885-022-09889-3)
Supplement: Supplementary file 1 — Additional file 1: Supplementary Table 1. Patient survival. Supplementary Fig. 1. Cox metanalysis of the impact of research factors on survival or risk rate. [file 12885_2022_9889_MOESM1_ESM.docx]

Supplementary Files

Supplementary Table

Supplementary Table 1. Patient survival

| Survival of all patients | | Liver-limited | *RAS* mutant | *RAS* WT | *BRAF*^V600E^ mutant | *RAS*/*BRAF*^V600E^ WT |
| --- | --- | --- | --- | --- | --- | --- |
|  | n=61 | n=15 | n=23 | n=31 | n=12 | n=19 |
| mPFS | 9.3 months | 10.0 moths | 9.7 months | 10.0 months | 9.3 months | NR |
| 1-year PFS | 35.0% | 40.0% | 40.3% | 32.1% | 38.1% | - |
| mOS | 32.2 moths | 34.7 months | 34.7 months | 18.9 months | 28.0 months | NR |
| 1-year OS | 84.6% | 93.3% | 90.1% | 82.0% | 74.1% | - |
| 2-year OS | 55.1% | 53.3% | 72.8% | 38.9% | 55.6% | - |
| 3-year OS | 38.6% | 42.7% | 48.5% | 34.0% | 33.3% | - |

OS, overall survival; PFS, progression-free survival; WT, wild-type

Supplementary Figure


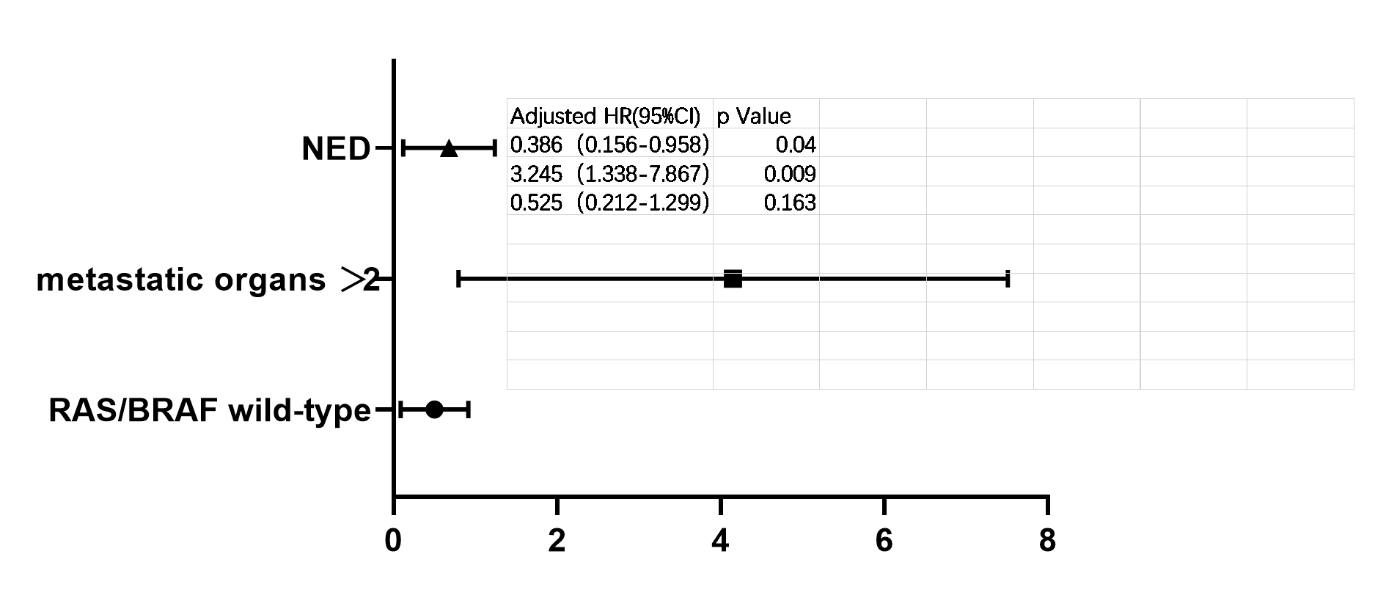


Supplementary Fig. 1. Cox regression analysis of the impact of research factors on survival or risk rate

NED, no evidence of disease
